# Evaluating National Trends in Bleeding Associated with Metabolic Bariatric Surgery over 7 Years

**DOI:** 10.1007/s11695-025-08231-7

**Published:** 2025-09-20

**Authors:** Tina Bharani, Divyansh Agarwal, Abdelrahman Nimeri, Thomas Tsai, Neil Ghushe, Malcolm Robinson, Talar Tatarian, Ali Tavakkoli, Eric Sheu

**Affiliations:** 1 Thomas Jefferson University Hospital, Philadelphia, PA USA; 2 Brigham and Women’s Hospital, Boston, MA USA; 3 Massachusetts General Hospital, Boston, MA USA

**Keywords:** Bleeding, Bleeding risk in MBS, Bleeding trends in MBS, Bleeding in SG, Bleeding in RYGB

## Abstract

**Background:**

Post-operative bleeding is a known complication after metabolic bariatric surgery (MBS). This study evaluates the national trends in the rates of bleeding, factors associated with bleeding, and impact of bleeding complication on other outcomes.

**Methods:**

MBSAQIP database from 2015 to 2021 was utilized to perform multivariable regression analysis of pre-operative factors associated with bleeding for all MBS, as well as gastric bypass (RYGB) and sleeve gastrectomy (SG) subsets. Propensity matching using pre-operative factors was performed for patients with and without a bleeding complication to compare peri-operative outcomes.

**Results:**

Rates of post-operative bleeding decreased overall from 1.01% in 2015 to 0.69% in 2021. RYGB (OR 2.08, *p* < 0.01) had a higher risk of bleeding compared to SG. Open surgical approach (OR 2.33, *p* < 0.01), therapeutic anticoagulation (OR 2.49, *p* < 0.01), renal insufficiency (OR 1.61, *p* < 0.01), and history of MI (OR 1.26, *p* < 0.01) were highly associated with bleeding. Pre-operative demographics associated with increased bleeding risk included older age (OR 1.16–1.31, *p* < 0.01), male gender (OR 1.10, *p* < 0.01) and Asian race (OR 1.47, *p* < 0.01). Staple line reinforcement (OR 0.76, *p* < 0.01) and oversewing (OR 0.79, *p* < 0.01) were protective against bleeding after SG. Bleeding was associated with 18 times higher risk of major complications (42.57% vs 2.33%, *p* < 0.01) and 8 times higher risk of death (1.06% vs 0.13%, *p* < 0.01).

**Conclusions:**

The risk of bleeding after MBS has decreased over the past 7 years. Patients suffering a bleeding complication have a markedly higher risk of major complications and death. Therefore, identifying methods to reduce post-operative bleeding should be a priority.

**Supplementary Information:**

The online version contains supplementary material available at 10.1007/s11695-025-08231-7.

## Introduction

Metabolic-bariatric surgery (MBS), the most effective intervention for patients with obesity and associated metabolic comorbidities, is safe with perioperative mortality rates ranging from 0.03 to 0.22% and serious adverse events of less than 6% [1–3]. Some of the more common post-MBS complications include venous thromboembolism, bleeding, anastomotic/staple line leak, and surgical site infection [1]. Post-operative bleeding is an ominous complication after MBS associated with significant morbidity and mortality [4]. The rate of post-operative bleeding after MBS has ranged from 1-4% in prior literature [5–7].

Bleeding can be intraluminal or intraabdominal [4, 8]. Intraluminal bleeding is typically related to the staple line in the early post-operative period or ulceration in later periods, while intraabdominal bleeding can be along the dissected planes, staple line, and ligated vessels, which can constitute a surgical emergency [8]. Some of the common symptoms and signs of bleeding after MBS include tachycardia, hypotension, decrease in hemoglobin and hematocrit, hematemesis/melena, and rarely, bowel obstruction secondary to intraluminal clot [4]. In many cases, bleeding is self-limited and resolves with supportive management [5]. However, some cases require reoperation and interventions and are associated with increased risk of morbidity and mortality [5].

While prior published literature has reported single institution experiences with bleeding and management approaches, little is known about the patient populations at higher risk of bleeding [4–7]. We sought to evaluate national trends in the rates of bleeding and factors associated with bleeding using the Metabolic and Bariatric Surgery Accreditation and Quality Improvement Program (MBSAQIP) database. Additionally, we examined the peri-operative outcomes in patients with a bleeding complication.

## Methods

### Study Design and Setting

The largest North-American database, MBSAQIP Public Use File (2015–2021), capturing the metabolic-bariatric operations at accredited centers in the USA and Canada was used for analysis [9].

### Participants

Patients undergoing primary MBS including sleeve gastrectomy (SG) (CPT 43775), Roux-en-Y gastric bypass (RYGB) (CPT 43644, 43846), and biliopancreatic diversion (BPD) (CPT 43845) were identified from 2015 to 2021 database. We utilized the CPT variable column denoting the principal operative procedure in the MBSAQIP database. Laparoscopic adjustable gastric band was excluded due to decreasing trends in utilization of this metabolic-bariatric approach. Endoscopic approaches and revisional MBS were also excluded. Endoscopic approaches were eliminated as they are still in experimental stages in the majority of institutions while revisional MBS was excluded due to higher overall rates of morbidity associated. Only laparoscopic, robotic, and open cases were included in the analysis.

### Variables

Bleeding as defined by MBSAQIP is transfusion of more than or equal to 1 unit of blood post-operatively, or readmission, reoperation, or intervention for bleeding. Therefore, all the patients who had bleeding complication as defined by the MBSAQIP criteria were included in the analysis. Pre-operative demographics including age, body mass index (BMI), sex and race, pre-operative cardiac, pulmonary, renal, hematologic, and endocrine comorbidities, American Society of Anesthesiologists (ASA) class, smoking status, surgical approach, and operative length were utilized to identify risk factors associated with bleeding. Post-operative outcomes compared between patients who had post-operative bleeding and those who did not included acute renal failure, cardiac arrest, myocardial infarction (MI), cerebrovascular accident (CVA), ventilation > 48 h, surgical site infections, sepsis, pulmonary embolism (PE), pneumonia (PNA), unplanned intubation and ICU admission, readmissions, reoperation, interventions, major complications, and mortality within 30 days. Major complications included reoperation, unplanned ICU admission, unplanned intubation, sepsis, pneumonia, organ space infection, MI, cardiac arrest, CVA, PE, and death within 30 days.

### Statistical Methods

Multivariable regression analysis of preoperative demographics and comorbidities in patients with post-operative bleeding was performed for all bariatric surgeries to identify factors associated with higher risk of bleeding. Sub-group analysis was then performed for SG and RYGB cohorts. Multivariable logistic regression was also performed on the SG subset from 2015 to 2019 to evaluate the association of bleeding with staple line reinforcement/oversewing. Data from the years 2020 and 2021 was excluded since the variable for staple line reinforcement/oversewing was not included in those two databases.

To compare the peri-operative outcomes between patients who had bleeding and those who did not, 1:1 propensity matching was performed using the nearest neighbor algorithm based on the generalized linear model distance. The variables that were utilized to match subjects included age, race, sex, history of MI, highest known BMI pre-operatively, hypertension (HTN), hyperlipidemia (HLD), gastroesophageal reflux disease (GERD), renal insufficiency, diabetes mellitus (DM), chronic obstructive pulmonary disease (COPD), obstructive sleep apnea (OSA), surgical approach (laparoscopic/robotic/open), type of surgery (SG, RYGB, BPD), ASA classification, history of deep vein thrombosis (DVT), hematocrit pre-operatively, venous stasis, dialysis, therapeutic anticoagulation, history of PE, IVC (inferior vena cava) filter, previous foregut surgery, and smoking status within 1 year pre-operatively. Diabetes was defined by two categories: insulin dependent (IDDM) and non-insulin dependent diabetes (NIDDM). Univariate analysis was then performed using the chi-square test for categorical variables and the non-parametric Mann Whitney U test for continuous data. All analyses were performed using R: A Language and Environment for Statistical Computing, version 4.0.3. [10].

## Results

**Fig. 1:**
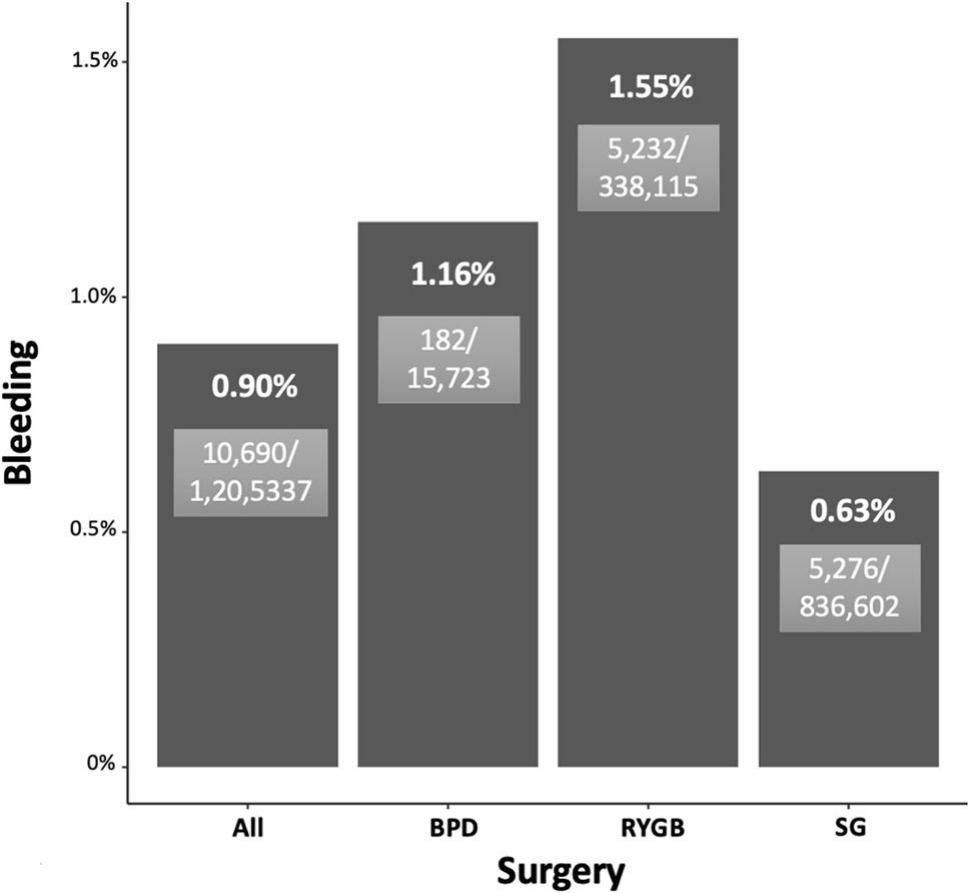
Rates of bleeding by type of surgery

Evaluating the rates of bleeding by type of MBS revealed highest rates associated with RYGB [5232/338,115 (1.55%)], followed by BPD [182/15,723 (1.16%)], and SG [5276/836,602 (0.63%) (0.15%)] (Fig. 1). The overall rates of bleeding associated with MBS have decreased over the past 7 years from 1.01% in 2015 to 0.69% (*p* < 0.01) in 2021 (Table 1). For SG, the rates have decreased from 0.71 to 0.53% (*p* < 0.01); and for RYGB, the rates have declined from 1.78 to 1.08% (*p* < 0.01) over the past 7 years (Table 1).

**Table 1:** Rates of bleeding from 2015-2021

	Overall	SG	RYGB
Year	Bleeding (N (%))	Bleeding (N (%))	Bleeding (N (%))
**2015**	1,500 (1.01%)	694 (0.71%)	785 (1.78%)
**2016**	1,504 (0.92%)	681 (0.60%)	790 (1.78%)
**2017**	1,609 (0.92%)	793 (0.63%)	787 (1.71%)
**2018**	1,754 (0.98%)	904 (0.71%)	807 (1.70%)
**2019**	1,794 (0.99%)	884 (0.70%)	870 (1.73%)
**2020**	1,184 (0.75%)	607 (0.56%)	554 (1.19%)
**2021**	1,368 (0.69%)	713 (0.53%)	639 (1.08%)
***p***-**value**	<0.01	<0.01	<0.01

Baseline demographics and comorbidities of patients with bleeding complications versus those who did not have a bleeding complication are shown in Supplementary Table 1. Multivariable logistic regression of pre-operative demographics and comorbidities associated with post-operative bleeding in patients undergoing MBS is shown in Table 2. Males are at 10% higher risk of bleeding compared to females. Asians are at 47% higher risk, and African Americans are at 6% higher risk of bleeding compared to whites undergoing MBS. Comorbidities that are highly associated with rates of bleeding included history of MI [OR 1.27 (1.11–1.44)], HTN [OR 1.22 (1.15, 1.28)], renal insufficiency [1.61 (1.35, 1.91)], venous thrombosis requiring therapy [1.23 (1.09, 1.40)], and IVC filter [1.29 (1.05, 1.57)]. Factors including therapeutic anticoagulation, open approach, and longer operative times are associated with more than twice the risk of bleeding, and pre-operative hematocrit < 35 has 61% higher risk of bleeding (Table 2). There is no significant difference in rates of bleeding according to the ASA classification, presence of HLD, history of PE, and venous stasis (Table 2). Additionally, no significant difference in the rates of bleeding was evident between laparoscopic and robotic approaches (Table 2).

**Table 2:** Multivariable logistic regression of pre-operative demographics and comorbidities associated with bleeding in patients undergoing bariatric surgery

Characteristic	OR^1^	95% CI^1^	p-value
**Age**			
20-40	Ref		
41-60	**1.16**	**1.09, 1.22**	**<0.01**
61-75	**1.31**	**1.21, 1.42**	**<0.01**
>75	1.29	0.80, 1.97	0.3
**Sex**			
Female	Ref		
Male	**1.10**	**1.04, 1.17**	**<0.01**
**Race**			
White	Ref		
American Indian or Alaska Native	0.91	0.65, 1.23	0.6
Asian	**1.47**	**1.14, 1.87**	**<0.01**
Black or African American	**1.06**	**1.00, 1.12**	**0.04**
**ASA Class**			
ASA I - Normal/Healthy	Ref		
ASA II - Mild systemic disease	1.29	0.75, 2.49	0.4
ASA III - Severe systemic disease	1.32	0.77, 2.55	0.4
ASA IV - Severe systemic disease threat to life	**1.77**	**1.02, 3.44**	**0.06**
**Highest BMI pre-operatively **			
35-39.9	Ref		
40-44.9	**0.90**	**0.85, 0.97**	**<0.01**
45-49.9	**0.80**	**0.74, 0.86**	**<0.01**
50-59.9	**0.75**	**0.70, 0.81**	**<0.01**
60 and above	**0.75**	**0.68, 0.83**	**<0.01**
History of MI	**1.27**	**1.11, 1.44**	**<0.01**
GERD requiring medication	**1.15**	**1.10, 1.20**	**<0.01**
Hypertension requiring medication	**1.22**	**1.15, 1.28**	**<0.01**
Hyperlipidemia	1.03	0.98, 1.09	0.2
Vein Thrombosis Requiring Therapy	**1.23**	**1.09, 1.40**	**<0.01**
Pre-Op Renal Insufficiency	**1.61**	**1.35, 1.91**	**<0.01**
Previous Surgery	**1.09**	**1.01, 1.17**	**0.03**
Current Smoker within 1 year	**1.13**	**1.05, 1.23**	**<0.01**
History of COPD	**1.28**	**1.12, 1.45**	**<0.01**
Anastomosis checked	**0.87**	**0.82, 0.92**	**<0.01**
Obstructive Sleep Apnea	**1.11**	**1.06, 1.16**	**<0.01**
Diabetes	**1.16**	**1.10, 1.22**	**<0.01**
History of PE	1.11	0.96, 1.28	0.2
Venous Stasis	1.12	0.94, 1.33	0.2
Dialysis	**1.39**	**1.08, 1.77**	**0.01**
Therapeutic Anticoagulation	**2.49**	**2.27, 2.71**	**<0.01**
IVC Filter	**1.29**	**1.05, 1.57**	**0.01**
**Surgical Approach**			
Laparoscopic	Ref		
Open	**2.33**	**1.74, 3.05**	**<0.01**
Robotic	0.99	0.90, 1.08	0.8
**Hematocrit**			
35-50	Ref		
<35	**1.61**	**1.48, 1.75**	**<0.01**
>50	0.97	0.79, 1.18	0.8
**Surgery**			
Sleeve	Ref		
BPD	**1.52**	**1.27, 1.81**	**<0.01**
RYGB	**2.08**	**1.97, 2.20**	**<0.01**
**Operative length (minutes)**			
<90	Ref		
90-179	**1.14**	**1.08, 1.20**	**<0.01**
180-269	**1.21**	**1.11, 1.33**	**<0.01**
270-359	**1.60**	**1.35, 1.90**	**<0.01**
360-479	**2.46**	**1.82, 3.24**	**<0.01**
480 and above	1.78	0.88, 3.17	0.07

^1^ *OR* Odds Ratio, *CI* Confidence Interval

Analysis of procedure-specific factors associated with bleeding for patients undergoing SG or RYGB revealed multiple high-risk factors that were common, including history of MI, renal insufficiency, COPD, therapeutic anticoagulation, and open approach. Specifically, in patients undergoing SG, there was no significant difference in the rates of bleeding by sex. African American patients also had a similar risk of bleeding compared to whites. Longer operative times were associated with up to 7 times higher risk of bleeding (Supplementary Table 2). Sub-analysis of patients undergoing SG from 2015 to 2019 showed that staple line reinforcement (OR 0.76, *p* < 0.01) and oversewing (OR 0.79, *p* < 0.01) were protective against bleeding after SG (Supplementary Table 2). Multivariable logistic regression of pre-operative factors among patients undergoing RYGB showed that males were at 18% higher risk of bleeding (Supplementary Table 3). Open approach was associated with 72% higher risk of bleeding, while robotic approach was associated with 13% lower risk of bleeding compared to laparoscopic approach (Supplementary Table 3).

Propensity match among pre-operative demographics and comorbidities was performed to elucidate the association between bleeding and post-operative complications. Matching resulted in well-balanced cohorts of 8478 patients each, in the bleeding and non-bleeding category, with absolute standardized mean difference<0.05. Patients in the bleeding cohort had significantly higher rates of cardiac, pulmonary, and renal complications post-operatively (Table 3). The rate of pulmonary embolism was significantly higher in the bleeding cohort compared to the non-bleeding cohort (1.13% vs 0.19%, *p* < 0.01) (Table 3). Similarly, the rates of organ space infection were 8 times higher in the bleeding cohort versus the non-bleeding cohort (2.84% vs 0.35%, *p* < 0.01) (Table 3). Bleeding was associated with 18-fold increase in major complications (42.57% vs 2.33%, *p* < 0.01), 6-fold increase in readmissions (27.19% vs 4.50%, *p* < 0.01) and 8-fold increase in mortality (1.06% vs 0.13%, *p* < 0.01) (Table 3).

**Table 3:** Comparison of post-operative outcomes within 30 days in matched cohorts of patients with and without bleeding complications

Characteristics	No Bleed (*N*=8478)	Bleeding (*N*=8478)	Significance
Acute Renal Failure	12 (0.1%)	136 (1.6%)	<0.01
Cardiac Arrest	8 (0.09%)	100 (1.18%)	<0.01
Myocardial Infraction	5 (0.06%)	47 (0.55%)	<0.01
CVA	1 (0.01%)	18 (0.21%)	<0.01
Ventilation >48 Hrs	12 (0.14%)	181 (2.13%)	<0.01
Deep Incisional SSI	11 (0.13%)	29 (0.34%)	<0.01
Superficial SSI	0 (0.00%)	2 (0.02%)	0.50
Organ Space Infection	30 (0.35%)	241 (2.84%)	<0.01
Sepsis	0 (0.00%)	8 (0.09%)	<0.01
Pulmonary Embolism	16 (0.19%)	96 (1.13%)	<0.01
PNA	0 (0.00%)	5 (0.06%)	0.06
UTI	0 (0.00%)	9 (0.11%)	<0.01
Unplanned Intubation	17 (0.20%)	307 (3.62%)	<0.01
Unplanned ICU Admission	67 (0.79%)	1,885 (22.23%)	<0.01
Wound Disruption	9 (0.11%)	46 (0.54%)	<0.01
Reoperation	119 (1.41%)	2,618 (30.88%)	<0.01
Intervention	131 (1.55%)	1,296 (15.29%)	<0.01
Readmission	380 (4.50%)	2,305 (27.19%)	<0.01
Major Complications	197 (2.33%)	3,609 (42.57%)	<0.01
Death	11 (0.13%)	90 (1.06%)	<0.01

## Discussion

Our study reveals the rate of bleeding after MBS has improved over the 7-year study period. Our study also reveals a steady decline in the rates of bleeding after MBS from 2015 to 2021, from 1.01 to 0.69%, respectively. Similarly, a declining trend in the rates of bleeding is seen for SG (from 0.71 to 0.53%) and RYGB (from 1.78 to 1.08%) specifically over the past 7 years (Table 1). However, patients with a bleeding complication have a significantly higher risk of post-operative complications and mortality. For example, the risk of readmission is 6 times higher in bleeding cohorts compared to those who did not have a bleeding complication (Table 3). Prior studies have supported similar findings, including increased length of stay (> 4 days in 87% of patients), higher risk of readmissions, and increased risk of significant post-operative morbidity due to bleeding after MBS [4, 11]. We recommend that patients who have a bleeding complication be followed more closely after discharge to mitigate post-operative complications and attempt to reduce readmissions. This can be accomplished through more frequent telehealth appointments and home hospital set-ups, which facilitate vitals monitoring and blood collection for labs at home [12].

Our analysis shows that the risk of major complications is 42.6% in the bleeding cohort versus 2.3% in the non-bleeding cohort (*p* < 0.01) and mortality is 1.06% versus 0.13% (*p* < 0.01) (Table 3). Additionally, the risk of pulmonary embolism is 6 times higher, and the rate of organ space infection is 8 times higher in bleeding cohorts. The higher risk of pulmonary embolism could perhaps be secondary to discontinuation of prophylactic anticoagulation in the setting of bleeding post-operatively. Emphasis should be placed on educating the patients to increase physical activity if prophylactic anticoagulation is discontinued. Additionally, it would be advisable to perform bilateral lower extremity ultrasounds at follow-up visits and weigh the utility of DVT filters. The increase in organ space infection, which is often a surrogate for anastomotic/staple line leaks, could be explained by ischemia secondary to low intravascular volume caused by bleeding. Alternatively, the increased risk of intra-abdominal hematoma infection, especially in the setting of interventions and reoperations, could also hamper the healing of anastomoses and staple lines. To our best knowledge, this is the first comprehensive study evaluating the repercussions of bleeding on major complications and mortality after MBS at the national level. Bleeding is associated with significantly worse patient outcomes and increased utilization of healthcare resources and expenditure. Thus, identification of high-risk patient population and adoption of strategies to minimize bleeding is of utmost importance.

Prior data has shown increased risk of bleeding associated with T2DM, HTN, history of renal insufficiency, and therapeutic anticoagulation [5, 13–15]. This is concordant with our analysis showing 16% higher risk of bleeding associated with T2DM, 61% higher risk with renal insufficiency, and 149% higher risk with therapeutic anticoagulation. Given the significantly higher risk of bleeding associated with patients on therapeutic anticoagulation, it is advisable to stop anticoagulation appropriately in the pre-operative period and consider consulting specialists for patients who have comorbidities slowing the elimination of anticoagulants. Additional risk factors associated with bleeding that were identified on multivariate analysis in our study include older age, male gender, Asian race, history of MI, GERD, COPD, and previous foregut surgery (Table 2). Open approach was associated with high risk of bleeding for both SG and RYGB (Supplementary Tables 2 and 3). There was no significant difference in bleeding associated with laparoscopic or robotic SG (Supplementary Tables 2), while robotic RYGB was associated with lower risk of bleeding compared to laparoscopic RYGB (Supplementary Tables 3). The lower risk of bleeding for RYGB with robotic approach as opposed to laparoscopic approach has also been evidenced in a prior MBSAQIP database study, likely secondary to improved visualization, increased utilization of staplers integrated in the robotic console over hand-sewn anastomosis, and surgeons being farther on the learning curve due to increased utilization of robotics [16]. We recommend further studies to evaluate the effect of laparoscopic versus robotic approaches to guide the surgeons to minimize the risk of bleeding for RYGB. Specifically for SG, staple line reinforcement and oversewing were protective against bleeding (Supplementary Tables 2). Recognition of factors associated with worse outcomes provides an opportunity to modify or optimize the patients pre-operatively. For instance, correction of iron-deficiency anemia with oral or intravenous iron during the prehabilitation period has shown to decrease transfusion requirements and improve clinical outcomes [17, 18].

In fact, evidence supports a decreasing rate of obesity-related comorbidities over time for patients undergoing MBS [19]. The overall decline in the rate of bleeding is driven more by RYGB than SG. This is an advancement compared to prior studies that have reported post-operative bleeding rates up to 3% associated with bariatric surgery [7, 13, 20, 21]. Increasing interest in prehabilitation with exercise and meal replacement programs leading to improvement in insulin resistance and cardiometabolic comorbidities may partially explain the decline in bleeding rates [7, 13, 20–23].

Some of the prior literature has suggested reduced rates of post-operative bleeding through various intra-operative interventions to detect bleeding such as increasing mean arterial pressure by 30% and reducing pneumoperitoneum to 8 mmHg in the last 15 minutes of the operation [24]. Vigorous control of blood pressure in the perioperative and post-operative period has also shown to minimize the risk of anastomotic bleeding [6]. Additionally, evidence supports reduced rates of bleeding associated with staple line reinforcement, as seen in our study [15, 25–27]. Contrary to prior evidence which shows no significant difference or increased risk of bleeding associated with oversewing, our study shows a 21% reduction in bleeding with oversewing (Supplementary Tables 2) [25, 27]. Given the retrospective nature of the study, one cannot establish a causal relationship between oversewing and bleeding. Additionally, the applicability of the findings related to oversewing and staple line reinforcement may be limited given the missing data in the years beyond 2019. However, it is possible that with increased experience over the years and improved techniques, metabolic-bariatric surgeons have minimized the tissue ischemia and insult associated with bleeding secondary to oversewing [27]. Additionally, various technical methods of anastomotic creation have also evolved over time including hand-sewn vs. stapled anastomosis (end-to-end anastomosis staplers vs linear staplers), staple reinforcement, new stapler technologies with varying stapler heights (e.g. tri-stapler, tissue thickness detectors with automated staple heights) and use of topical thrombotic agents (gel-foam, tisseel, vistaseal). While this data is not captured in the MBSAQIP database, changes in the techniques utilized for anastomosis creation may influence bleeding risk in varying degrees. Evidence supports lower risk of intra-luminal bleeding associated with use of linear staplers in creation of gastrojejunostomy compared to circular staplers [7, 14]. Additionally, differences in staple heights have also shown to affect the risk of bleeding with 3.5 mm height providing optimal tissue compression with adequate hemostasis as compared to 4.8 mm height [28, 29].

Our study is not without limitations. Given the retrospective nature of the work, it does not allow for determination of causal relationship between bleeding and pre-operative factors that were identified as high risk. Furthermore, some of the confounders such as differences in surgeon techniques and tissue handling, difference in staple heights used, post-operative choice and dose of prophylactic anticoagulation, threshold for blood transfusion, and presence of pre-operative coagulation disorders are not captured in the MBSAQIP database. Additionally, the comparison between laparoscopic and robotic approaches lacks confounders such as surgeon experience and selection bias. While the MBSAQIP definition of bleeding was comprehensive to include anyone who received blood transfusion after MBS or had any readmission, reoperation, or intervention secondary to bleeding, some patients with self-contained bleeding may have been managed conservatively with no transfusions due to provider preference. Moreover, we are unable to distinguish the severity of bleeding leading to worsening post-operative complications or requiring interventions due to the lack of relevant parameters in the MBSAQIP database. Lastly, the findings of our study are focused on the database from North America, which limits its generalization globally. However, ours is the largest study to date capturing the bleeding experience at North American bariatric surgical centers of excellence over the past 7 years.

## Conclusion

The risk of bleeding after MBS has decreased in the past 7 years, with an overall rate of < 1% and a higher risk after RYGB compared to SG. Patients suffering a bleeding complication have a markedly higher risk of major complications and death. Therefore, identifying methods to reduce post-operative bleeding should remain a priority and may improve overall outcomes of MBS.

## Supplementary Information

Below is the link to the electronic supplementary material.

Supplementary Material 1 (DOCX 18.5 KB)

Supplementary Material 2 (DOCX 19.6 KB)

Supplementary Material 3 (DOCX 19.8 KB)

## Data Availability

No datasets were generated or analysed during the current study.
