# Supplementary material for: Evaluating National Trends in Bleeding Associated with Metabolic Bariatric Surgery over 7 Years
Source: Obes Surg. 2025 Sep 20;35(10):4079–86. doi: 10.1007/s11695-025-08231-7 (PMC12540593; doi:10.1007/s11695-025-08231-7)
Supplement: Supplementary file 1 — Supplementary Material 1 (DOCX 18.5 KB) [file 11695_2025_8231_MOESM1_ESM.docx]

**Supplementary Table 1: Preoperative demographics and comorbidities of patients with bleeding complication versus those with no bleeding complication (2015-2021)**

| Characteristics | No bleeding (1,179,290) | Bleeding (10,690) | Significance |
| --- | --- | --- | --- |
| Age | 44 (35, 53) | 48 (39, 57) | <0.01 |
| Highest pre-op BMI | 45.35 (41.20, 51.21) | 44.70 (40.55, 50.85) | <0.01 |
| **Sex** |  |  | <0.01 |
| Female | 952,273 (81%) | 8,194 (77%) |  |
| Male | 227,220 (19%) | 2,494 (23%) |  |
| **Race** |  |  | 0.03 |
| American Indian or Alaska Native | 5,289 (0.5%) | 53 (0.5%) |  |
| Asian | 6,221 (0.6%) | 78 (0.8%) |  |
| Black or African American | 220,668 (21%) | 2,043 (21%) |  |
| White | 824,925 (78%) | 7,499 (78%) |  |
| **ASA CLASS** |  |  | <0.01 |
| ASA I - Normal/Healthy | 3,467 (0.3%) | 22 (0.2%) |  |
| ASA II - Mild systemic disease | 246,533 (21%) | 1,848 (17%) |  |
| ASA III - Severe systemic disease | 884,040 (75%) | 8,148 (76%) |  |
| ASA IV - Severe systemic disease threat to life | 41,652 (3.5%) | 639 (6.0%) |  |
| Hyperlipidemia | 270,876 (23%) | 3,502 (33%) | <0.01 |
| **Surgical Approach** |  |  | <0.01 |
| Laparoscopic | 1,101,211 (93%) | 9,892 (93%) |  |
| Open | 1,910 (0.2%) | 73 (0.7%) |  |
| Robotic | 76,629 (6.5%) | 725 (6.8%) |  |
| HTN | 544,711 (46%) | 6,298 (59%) | <0.01 |
| Renal Insufficiency | 6,899 (0.6%) | 209 (2.0%) | <0.01 |
| History of MI | 13,659 (1.2%) | 317 (3.0%) | <0.01 |
| GERD | 370,696 (31%) | 4,425 (41%) | <0.01 |
| OSA | 437,525 (37%) | 4,836 (45%) | <0.01 |
| COPD | 17,191 (1.5%) | 324 (3.0%) | <0.01 |
| Smoker | 91,335 (7.7%) | 886 (8.3%) | 0.04 |
| Pre-op DVT on therapy | 19,757 (1.7%) | 437 (4.1%) | <0.01 |
| Diabetes |  |  | <0.01 |
| NIDDM | 200,164 (17%) | 2,248 (21%) |  |
| IDDM | 88,893 (7.5%) | 1,372 (13%) |  |
| Venous stasis | 9,955 (0.8%) | 178 (1.7%) | <0.01 |
| Dialysis | 3,532 (0.3%) | 108 (1.0%) | <0.01 |
| Therapeutic anticoagulation | 32,364 (2.7%) | 1,023 (9.6%) | <0.01 |
| Previous obesity/ foregut surgery | 93,972 (8.0%) | 1,368 (13%) | <0.01 |
| History of PE | 14,770 (1.3%) | 317 (3.0%) | <0.01 |
| IVC filter | 5,601 (0.5%) | 133 (1.2%) | <0.01 |
| Pre-op hematocrit | 40.9 (38.6, 43.3) | 40.7 (38.0, 43.3) | <0.01 |
| Mortality within 30 days | 925 (<0.1%) | 112 (1.0%) | <0.01 |
