# Supplementary material for: Evaluating National Trends in Bleeding Associated with Metabolic Bariatric Surgery over 7 Years
Source: Obes Surg. 2025 Sep 20;35(10):4079–86. doi: 10.1007/s11695-025-08231-7 (PMC12540593; doi:10.1007/s11695-025-08231-7)
Supplement: Supplementary file 2 — Supplementary Material 2 (DOCX 19.6 KB) [file 11695_2025_8231_MOESM2_ESM.docx]

**Supplementary Table 2: Multivariable logistic regression of pre-operative demographics and comorbidities associated with bleeding in patients undergoing SG from 2015-2021**

| **Characteristic** | **OR** | **95% CI** | **p-value** |
| --- | --- | --- | --- |
| **Age** |  |  |  |
| 20-40 | Ref |  |  |
| 41-60 | **1.24** | **1.14, 1.34** | **<0.01** |
| 61-75 | **1.46** | **1.30, 1.63** | **<0.01** |
| >75 | 1.07 | 0.53, 1.91 | 0.80 |
| **Sex** |  |  |  |
| Female | Ref |  |  |
| Male | 1.03 | 0.95, 1.11 | 0.50 |
| **Race** |  |  |  |
| White | Ref |  |  |
| American Indian or Alaska Native | 1.36 | 0.88, 1.99 | 0.14 |
| Asian | **1.55** | **1.08, 2.14** | **0.01** |
| Black or African American | 0.99 | 0.91, 1.07 | 0.80 |
| Native Hawaiian or Other Pacific Islander | 0.81 | 0.37, 1.51 | 0.50 |
| **ASA Class** |  |  |  |
| ASA I - Normal/Healthy | Ref |  |  |
| ASA II - Mild systemic disease | 1.36 | 0.67, 3.44 | 0.50 |
| ASA III - Severe systemic disease | 1.44 | 0.71, 3.63 | 0.40 |
| ASA IV - Severe systemic disease threat to life | 2.06 | 1.00, 5.22 | 0.08 |
| **Highest BMI pre-op** |  |  |  |
| 35-39.9 | Ref |  |  |
| 40-44.9 | **0.87** | **0.79, 0.95** | **<0.01** |
| 45-49.9 | **0.78** | **0.71, 0.87** | **<0.01** |
| 50-59.9 | **0.72** | **0.65, 0.80** | **<0.01** |
| 60 and above | **0.72** | **0.62, 0.83** | **<0.01** |
| HLD | 1.06 | 0.98, 1.14 | 0.20 |
| History of MI | 1.13 | 0.92, 1.36 | 0.20 |
| GERD requiring medications | **1.20** | **1.12, 1.29** | **<0.01** |
| HTN requiring medications | **1.28** | **1.19, 1.38** | **<0.01** |
| Pre-op VTE requiring therapy | **1.22** | **1.01, 1.45** | **0.04** |
| Renal insufficiency | **1.80** | **1.43, 2.24** | **<0.01** |
| Previous obesity/ foregut surgery | 1.04 | 0.91, 1.19 | 0.50 |
| Smoker | 1.11 | 0.99, 1.24 | 0.07 |
| COPD | **1.41** | **1.18, 1.68** | **<0.01** |
| Diabetes | **1.15** | **1.07, 1.24** | **<0.01** |
| History of PE | 1.04 | 0.84, 1.28 | 0.70 |
| Venous stasis | 1.20 | 0.93, 1.52 | 0.14 |
| Dialysis | 1.31 | 0.97, 1.75 | 0.07 |
| Therapeutic anticoagulation | **2.58** | **2.28, 2.91** | **<0.01** |
| IVC filter | 1.11 | 0.81, 1.49 | 0.50 |
| OSA | **1.13** | **1.05, 1.21** | **<0.01** |
| Anastomosis checked | **0.79** | **0.74, 0.85** | **<0.01** |
| Surgical approach |  |  |  |
| Laparoscopic | Ref |  |  |
| Open | **10.5** | **4.97, 20.3** | **<0.01** |
| Robotic | 1.09 | 0.96, 1.22 | 0.20 |
| HCT |  |  |  |
| 35-50 | Ref |  |  |
| <35 | **1.76** | **1.56, 1.97** | **<0.01** |
| >50 | 1.08 | 0.82, 1.40 | 0.60 |
| Operative length (mins) |  |  |  |
| <90 | — | — |  |
| 90-179 | **1.24** | **1.15, 1.33** | **<0.01** |
| 180-269 | **2.11** | **1.74, 2.54** | **<0.01** |
| 270-359 | **4.58** | **3.01, 6.66** | **<0.01** |
| 360-479 | **7.45** | **3.73, 13.5** | **<0.01** |
| 480 and above | **2.95** | **1.03, 6.59** | **0.02** |
| Oversew* | **0.79** | **0.72, 0.87** | **<0.01** |
| Staple line reinforcement* | **0.76** | **0.71, 0.82** | **<0.01** |

*Multivariable logistic regression for patients undergoing SG during the years 2015-2019
