# Supplementary material for: Evaluating National Trends in Bleeding Associated with Metabolic Bariatric Surgery over 7 Years
Source: Obes Surg. 2025 Sep 20;35(10):4079–86. doi: 10.1007/s11695-025-08231-7 (PMC12540593; doi:10.1007/s11695-025-08231-7)
Supplement: Supplementary file 3 — Supplementary Material 3 (DOCX 19.8 KB) [file 11695_2025_8231_MOESM3_ESM.docx]

**Supplementary Table 3: Multivariable logistic regression of pre-operative demographics and comorbidities associated with bleeding in patients undergoing RYGB from 2015-2021**

| **Characteristic** | **OR** | **95% CI** | **p-value** |
| --- | --- | --- | --- |
| Age |  |  |  |
| 20-40 | Ref |  |  |
| 41-60 | 1.06 | 0.98, 1.15 | 0.15 |
| 61-75 | **1.15** | **1.02, 1.29** | **0.02** |
| >75 | 1.61 | 0.79, 2.90 | 0.15 |
| Sex |  |  |  |
| Female | Ref |  |  |
| Male | **1.18** | **1.09, 1.28** | **<0.01** |
| Race |  |  |  |
| White | Ref |  |  |
| American Indian or Alaska Native | 0.60 | 0.34, 0.98 | 0.06 |
| Asian | **1.45** | **1.00, 2.03** | **0.04** |
| Black or African American | **1.15** | **1.06, 1.25** | **0.01** |
| Native Hawaiian or Other Pacific Islander | **1.65** | **1.06, 2.45** | **0.02** |
| ASA Class |  |  |  |
| ASA I - Normal/Healthy | Ref |  |  |
| ASA II - Mild systemic disease | 1.14 | 0.52, 3.20 | 0.80 |
| ASA III - Severe systemic disease | 1.11 | 0.51, 3.12 | 0.80 |
| ASA IV - Severe systemic disease threat to life | 1.37 | 0.62, 3.88 | 0.50 |
| Highest BMI pre-op |  |  |  |
| 35-39.9 | Ref |  |  |
| 40-44.9 | 0.95 | 0.86, 1.05 | 0.30 |
| 45-49.9 | **0.81** | **0.73, 0.90** | **<0.01** |
| 50-59.9 | **0.79** | **0.71, 0.87** | **<0.01** |
| 60 and above | **0.75** | **0.65, 0.86** | **<0.01** |
| History of MI | **1.38** | **1.14, 1.66** | **<0.01** |
| GERD requiring medications | **1.09** | **1.02, 1.16** | **0.01** |
| HTN requiring medications | **1.13** | **1.05, 1.22** | **<0.01** |
| HLD | 1.02 | 0.94, 1.11 | 0.60 |
| Pre-op VTE requiring therapy | **1.25** | **1.05, 1.49** | **0.01** |
| Renal insufficiency | **1.39** | **1.03, 1.83** | **0.02** |
| Previous obesity/ foregut surgery | **1.11** | **1.01, 1.22** | **0.03** |
| Smoker | **1.18** | **1.04, 1.32** | **<0.01** |
| COPD | 1.13 | 0.93, 1.37 | 0.20 |
| Diabetes | **1.18** | **1.10, 1.27** | **<0.01** |
| History of PE | 1.20 | 0.98, 1.46 | 0.07 |
| Venous stasis | 1.01 | 0.77, 1.30 | >0.90 |
| Dialysis | 1.20 | 0.72, 1.90 | 0.50 |
| Therapeutic anticoagulation | **2.42** | **2.12, 2.76** | **<0.01** |
| IVC filter | 1.31 | 0.97, 1.74 | 0.06 |
| OSA | **1.09** | **1.02, 1.17** | **0.01** |
| Surgical approach |  |  |  |
| Laparoscopic | Ref |  |  |
| Open | **1.72** | **1.16, 2.44** | **<0.01** |
| Robotic | **0.87** | **0.76, 0.99** | **0.04** |
| HCT |  |  |  |
| 35-50 | Ref |  |  |
| <35 | **1.46** | **1.28, 1.65** | **<0.01** |
| >50 | 0.85 | 0.61, 1.15 | 0.30 |
| Operative Length (mins) |  |  |  |
| <90 | Ref |  |  |
| 90-179 | 0.98 | 0.91, 1.06 | 0.70 |
| 180-269 | 0.99 | 0.89, 1.10 | 0.80 |
| 270-359 | **1.29** | **1.05, 1.57** | **0.01** |
| 360-479 | **1.90** | **1.33, 2.62** | **<0.01** |
| 480 and above | 1.23 | 0.44, 2.71 | 0.60 |
